# Whole genome analysis of hepatitis B virus before and during long-term therapy in chronic infected patients: Molecular characterization, impact on treatment and liver disease progression

**DOI:** 10.3389/fmicb.2022.1020147

**Published:** 2022-10-17

**Authors:** Zeineb Belaiba, Kaouther Ayouni, Mariem Gdoura, Wafa Kammoun Rebai, Henda Touzi, Amel Sadraoui, Walid Hammemi, Lamia Yacoubi, Salwa Abdelati, Lamine Hamzaoui, Mohamed Msaddak Azzouz, Anissa Chouikha, Henda Triki

**Affiliations:** ^1^Laboratory of Clinical Virology, WHO Reference Laboratory for Poliomyelitis and Measles in the Eastern Mediterranean Region, Pasteur Institute of Tunis, University Tunis El Manar (UTM), Tunis, Tunisia; ^2^Research Laboratory “Virus, Vectors and Hosts: One Health Approach and Technological Innovation for a Better Health,” LR20IPT02, Pasteur Institute of Tunis, University Tunis El Manar (UTM), Tunis, Tunisia; ^3^Laboratory of Biomedical Genomics and Oncogenetics (LR16IPT05), Pasteur Institute of Tunis, University Tunis El Manar (UTM), Tunis, Tunisia,; ^4^Department of Gastroenterology, Polyclinic of CNSS, Sousse, Tunisia; ^5^Department of Gastroenterology, Hospital of Tahar Maamouri, Nabeul, Tunisia

**Keywords:** HBV, antiviral resistance, liver cirrhosis, PCR, whole genome, Sanger sequencing, hepatocellular carcinoma

## Abstract

Hepatitis B virus (HBV) infection remains a serious public health concern worldwide despite the availability of an efficient vaccine and the major improvements in antiviral treatments. The aim of the present study is to analyze the mutational profile of the HBV whole genome in ETV non-responder chronic HBV patients, in order to investigate antiviral drug resistance, immune escape, and liver disease progression to Liver Cirrhosis (LC) or Hepatocellular Carcinoma (HCC). Blood samples were collected from five chronic hepatitis B patients. For each patient, two plasma samples were collected, before and during the treatment. Whole genome sequencing was performed using Sanger technology. Phylogenetic analysis comparing the studied sequences with reference ones was used for genotyping. The mutational profile was analyzed by comparison with the reference sequence M32138. Genotyping showed that the studied strains belong to subgenotypes D1, D7, and D8. The mutational analysis showed high genetic variability. In the RT region of the polymerase gene, 28 amino acid (aa) mutations were detected. The most significant mutations were the pattern rtL180M + rtS202G + rtM204V, which confer treatment resistance. In the S gene, 35 mutations were detected namely sP120T, sT126S, sG130R, sY134F, sS193L, sI195M, and sL216stop were previously described to lead to vaccine, immunotherapy, and/or diagnosis escape. In the C gene, 34 mutations were found. In particular, cG1764A, cC1766G/T, cT1768A, and cC1773T in the BCP; cG1896A and cG1899A in the precore region and cT12S, cE64D, cA80T, and cP130Q in the core region were associated with disease progression to LC and/or HCC. Other mutations were associated with viral replication increase including cT1753V, cG1764A/T, cC1766G/T, cT1768A, and cC1788G in the BCP as well as cG1896A and cG1899A in the precore region. In the X gene, 30 aa substitutions were detected, of which substitutions xT36D, xP46S, xA47T, xI88F, xA102V, xI127T, xK130M, xV131I, and xF132Y were previously described to lead to LC and/or HCC disease progression. In conclusion, our results show high genetic variability in the long-term treatment of chronic HBV patients causing several effects. This could contribute to guiding national efforts to optimize relevant HBV treatment management in order to achieve the global hepatitis elimination goal by 2030.

## Introduction

Hepatitis B virus (HBV) infection remains a serious public health concern worldwide despite the availability of an efficient vaccine and the major improvements in antiviral treatments. The World Health Organization (WHO) estimates that, in 2021, approximately 296 million persons are chronic HBV carriers. Among them, 820,000 represent a high risk of mortality caused by developing progressive liver diseases including hepatocellular carcinoma (HCC) and liver cirrhosis (LC) (WHO, 2021).

The genome of HBV is a circular DNA partially double-stranded of 3.2 kb and classified into 10 genotypes from A to J (Sunbul, 2014). It is organized into four main open overlapped reading frames (ORFs; pre-S1/pre-S2/S, pre-C/C, P, and X), encoding several proteins including the surface proteins S, M, and L holding the HBs antigen (HBsAg), the precore/core proteins holding HBeAg and HBcAg antigens, the polymerase (P), and the X protein holding the antigen HBxAg. Thus, mutations that occur in one gene can result in significant changes in the other overlapping genes.

Long-term treatment of HBV chronic patients with the available antiviral molecules can lead to the emergence of mutations throughout the whole genome. Mutations that occur within the reverse transcriptase (RT) domain of the P gene, target of antiviral treatment, may lead to treatment failure (Locarnini and Mason, 2006). Potential resistance-related mutations are grouped into 4 categories, primary mutations (category 1) could reduce antiviral susceptibility and HBV replication fitness. Secondary/compensatory mutations (category 2) developed subsequently and could restore functional defects in the RT activity of HBV caused by primary mutations. Putative antiviral resistance mutations (category 3) were reported as possible drug-resistant mutations but not verified experimentally and may be related to prolonged treatment or replication compensation. Pre-treatment mutations (category 4) could be found among treatment-naive patients but their role in antiviral treatment resistance has not been elucidated (Liu et al., 2010; Ciftci et al., 2014).

Moreover, mutations that emerge throughout a prolonged therapy could affect not only the RT region (Locarnini and Mason, 2006) but also the different overlapping genes. Therefore, such variations might result in hepatitis B immunoglobulin (HBIG) therapy escape, vaccine escape, misdiagnosis, and immune escape. They also could enhance viral replication capacity and viral persistence leading to the progression of severe liver diseases such as HCC or LC (Sheldon et al., 2006; Sheldon, 2008; Rajoriya et al., 2017).

On the other hand, it has been found that the presence of pre-existing naturally occurring mutations in treatment-naive patients may influence the efficacy of antiviral treatments. Therefore, knowledge of the mutational profile by whole genome sequencing of the HBV genome, for chronically infected patients, is of great interest for a complete diagnosis toward an efficient therapy scheme.

For HBV chronic patients in Tunisia, the national therapeutic schema is based on Entecavir (ETV) as a first-line of HBV treatment, and it is fully covered by the National Health Insurance Fund (NHIF), in case of resistance, Tenofovir disoproxil fumarate (TDF), is recommended alone or combined to ETV. However, the TDF is not covered by the NHIF.

The aim of the present study is to analyze the mutational profile through the HBV whole genome in ETV non-responder chronic HBV patients, in order to investigate antiviral drug resistance, immune escape, and liver disease progression to LC or HCC.

## Materials and methods

### Patients and samples

HBV chronic patients with quantifiable viral load and suspected to be ETV non-responders after viral breakthrough were included in the study. Blood samples were collected from five chronic hepatitis B patients investigated during the routine diagnostic activity of the Laboratory of Clinical Virology in Pasteur Institute of Tunis. For each included patient two plasma samples were collected: one before treatment as part of the pre-treatment diagnostic, and one during the treatment upon request of the treating physician. The period separating the second sample from the date of treatment beginning ranging between 14 and 72 months depending on the time of the viral breakthrough for each patient. Virological and clinical data are shown in Table 1.

**Table 1:** Virological, treatment molecules, and treatment duration data for the five Tunisian chronic HBV infected patients before and during therapy.

Patients	Gender	Age	Viral load (UI/ml)/ year		Date of treatment beginning	Treatment duration (months)	Clinical status	Treatment molecules	Subgenotype	HBsAg	HBeAg
			Before treatment	During treatment							
Patient 1	Male	53	>1,1.10^8^/2012	8,85.10^5^/2018	10/2012	72	CHB^+^	ETV^*^	D7	+	+
Patient 2	Male	N.A	1,85.10^7^/2011	6,79.10^4^/2017	04/2012	58	HCC^++^ (Deceased in 2020)	ETV^*^ and TDF^**^	D1	+	+
Patient 3	Male	N.A	2,38.10^6^/2006	4,74.10^6^/2016	12/2010	68	CHB^+^	ETV^*^ and TDF^**^	D1	+	+
Patient 4	Female	33	>1,1.10^8^/2016	5,89.10^3^/2018	02/2017	14	CHB^+^	ETV^*^	D7	+	+
Patient 5	Female	23	3,57.10^6^/2012	9,05.10^2^/2014	11/2012	16	CHB^+^	ETV^*^	D8	+	+

N.A, not available.

+ CHB, Chronic HBV Infection.

++ HCC, Hepatocellular Carcinoma.

* ETV, Entecavir.

** TDF, tenofovir disoproxil fumarate.

### Methods

#### DNA extraction, amplification, and sequencing

**Figure 1:**
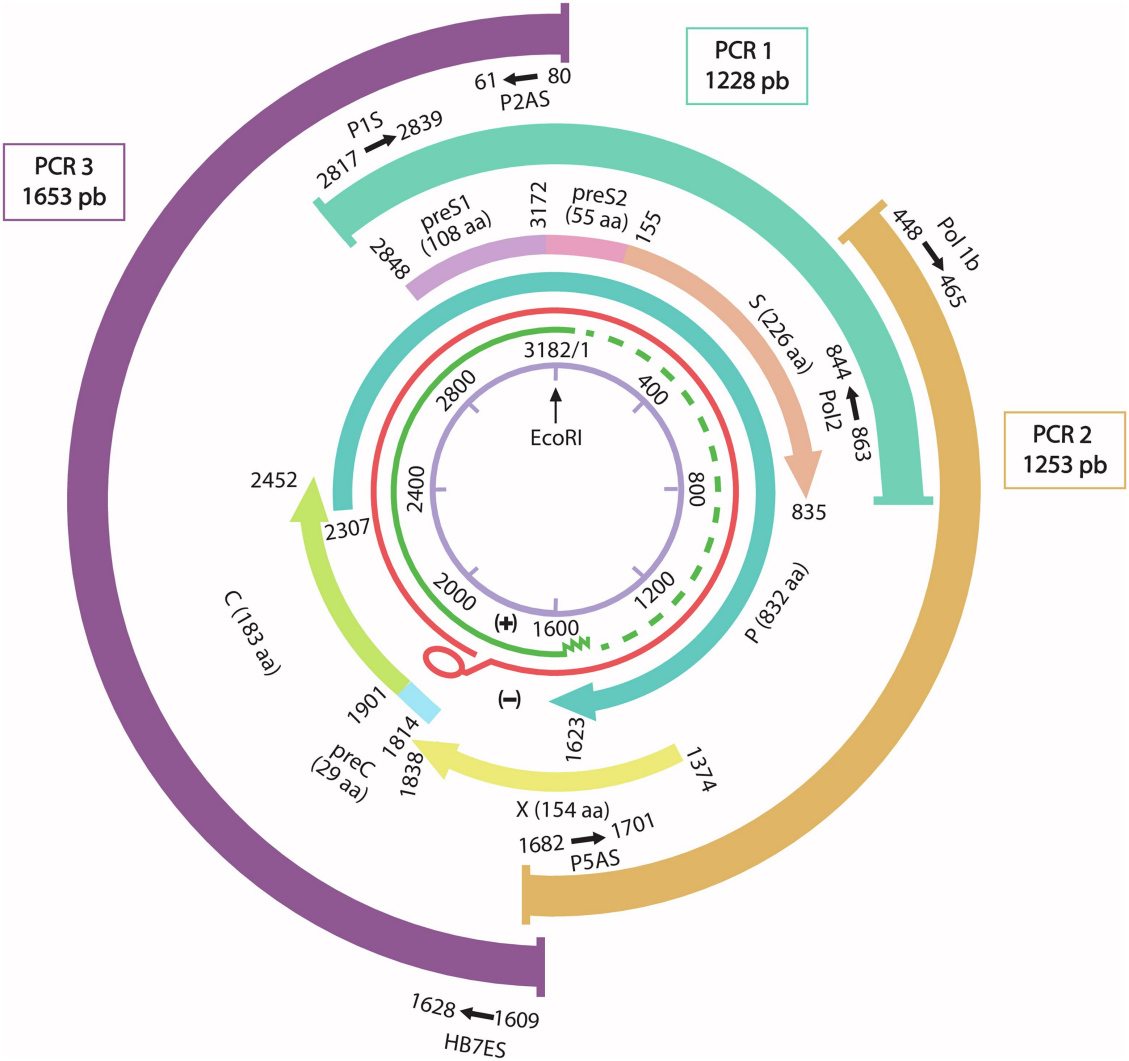
Representation of the HBV whole genome with the three overlapping PCR products and their primers sets positions. From inside to outside: schematic representation of the HBV genome with the EcoR1 restriction enzyme site representing nucleotide number 1. In green the incomplete positive strand, in Red the complete negative strand of the HBV genome. The overlapping genes P: Polymerase gene, S: Surface gene, C: capsid gene and X gene. Three overlapping fragments covering the whole genome are represented with the used primers P1S-Pol2 for PCR1 (1,228 nt), Pol1b-P5as for PCR2 (1,253 bp), and P2AS-HB7ES for PCR3 (1,653 bp).

DNA was extracted from 200 μl of plasma using the Qiagen QIAamp® DNA extraction kit (QIAGEN® Inc., Hilden, Germany) according to the manufacturer’s instructions. Three pairs of primers previously described by Chekaraou et al. (2010) were used to amplify 3 overlapping amplicons of 1,228-bp (nt 2,817–863), 1,253 bp (nt 448–1,701), and 1,653 bp (nt 1,609–80) covering the whole HBV genome as shown in Figure 1.

PCR reactions were performed in 50 μl of reaction mixture containing 1X polymerase buffer, 1.5 mM MgCl2, 0.2 mM dNTPs, 0.2 μM of each primer, 1.25 U of Taq Core MP® (Applied Biosystems) and nuclease-free water. The amount of DNA extract added varied between 10 to 35 μl depending on the viral load. PCR cycling was as follows: 94°C for 5 min, 40 cycles (94°C for 1 min, 56°C/57°C/62.5°C for regions 1, 2, and 3, respectively, 72°C for 1 min) with a final extension step at 72°C for 10 min. PCR products were analyzed by electrophoresis on 1% agarose gels stained with 1,25X of Red gel™ dye Nucleic Acid (Biotium®) and visualized by UV transilluminator.

The purified template DNA was sequenced using a BigDye Terminator Ready Reaction Cycle Sequencing Kit (Applied Biosystems) using the same primers pairs on an ABI Prism 3130 Genetic Analyzer (Applied Biosystems).

#### HBV genome assembly, genotyping, and subtyping

The obtained overlapping sequences were then assembled using BLAST multiple sequences software by comparison with a reference sequence (M32138).^1^ The generated final sequences were submitted to Genbank under accession numbers: MT591274-MT591281 and OP121186.

Sequence alignment was performed with MAFFT online server using default parameters^2^ by comparing the obtained genomic sequences with 58 reference sequences representing the 10 HBV genotypes (A–J) and their corresponding subgenotypes. The resulting alignment was used to build a maximum likelihood phylogenetic tree using the IQ-TREE web server, supported by 1,000 bootstrap replicates.^3^ The phylogenetic tree was then visualized using Figtree software.^4^ The tree was rooted using the midpoint rooting method. Genotypes were also confirmed by the National Center for Biotechnology Information’s (NCBI) E-genotype online software.^5^ HBV subtypes were inferred from sequences of the S gene by identifying amino acids (aa) at positions 122, 160, 127, 140, and 159 according to an algorithm previously described (Purdy et al., 2006).

#### Mutation analysis

Mutational profiles of the nucleotide or amino acid sequences were determined by comparing each gene (P, S, C, and X) before and during treatment with the corresponding reference sequence using Mega 7.026 (Kumar et al., 2016). Mutations’ impacts on treatment, immune response, and liver disease progression were analyzed based on the literature.

## Results

### HBV whole genome assembly

Whole genome sequences were obtained before and during treatment for 3 patients (1, 2, and 3). For the two remaining patients (4 and 5) we succeeded to obtain the whole genome before treatment. During treatment, the obtained sequence of patient 4 was lacking 408 bp (from nucleotide 45 to nt 453) and for patient 5 we could not be able to amplify the HBV genome which could be due to the low viral load.

### HBV genotyping and subtyping

**Figure 2:**
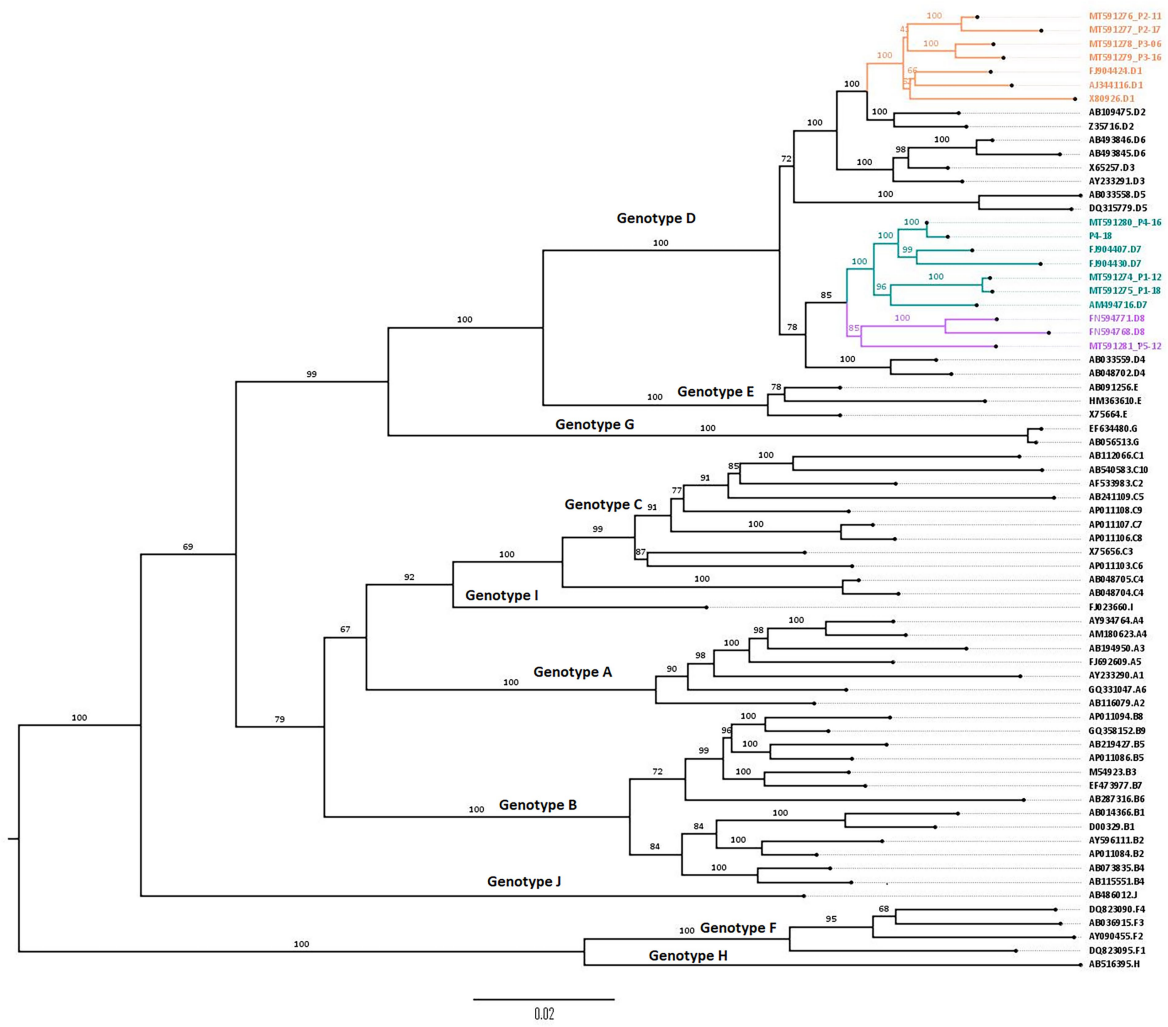
Phylogenetic tree constructed using the maximum likelihood method, showing D1, D7, and D8 subgenotypes from 5 Tunisian HBV patients. Phylogenetic tree of obtained genomic sequences with 58 reference sequences representing the 10 HBV genotypes (A–J) and their corresponding subgenotypes. The tree was constructed using the maximum likelihood method using the IQ tree web server and visualized by FigTree. Topology was supported by 1,000 bootstrap replicates. The tree was rooted using the midpoint rooting method.

Phylogenetic analysis (Figure 2) showed that all the sequences belong to genotype D. Subgenotyping showed that patients 1 and 4 were infected with subgenotype D7; patients 2 and 3 with D1 and patient 5 with D8, supported by high bootstrap values: 100, 100, and 85, respectively.

Subtyping showed that the studied HBV strains belonged to the ayw2 subtype based on Arg122, Lys160, Pro127, Tyr140, and Gly159 positions.

### Genetic variability in the P, S, C, and X genes

#### Mutational profile of the RT region in the polymerase gene

The mutational analysis of the RT region revealed a total of 28 aa substitutions ranging between 7 and 12 per patient, among them, several potential resistance-related mutations were detected. Primary mutations (category 1), rtS202G and rtM204V, occurred in patients 2 and 3 during treatment. Secondary/compensatory mutations (category 2), were found in 8 aa replacements; 5 were detected before treatment (rtL91I and rtT128N in patient 2; rtQ149K and rtP237T in patients 1, 4, and 5; rtQ267H in patients 4 and 5) and 3 changes emerged during treatment (rtL180M in patients 2 and 3; rtQ215S and rtF221Y in patient 2).

Three putative antiviral resistance mutations (category 3) were detected: rtR153W in treatment-naïve patients 1, 4, and 5 as well as rtD134E and rtC256S during treatment in patients 3 and 1, respectively. Three pre-treatment mutations (category 4) were also found: rtR110G and rtI266R in patient 1 and rtD263E in patient 5.

**Figure 3:**
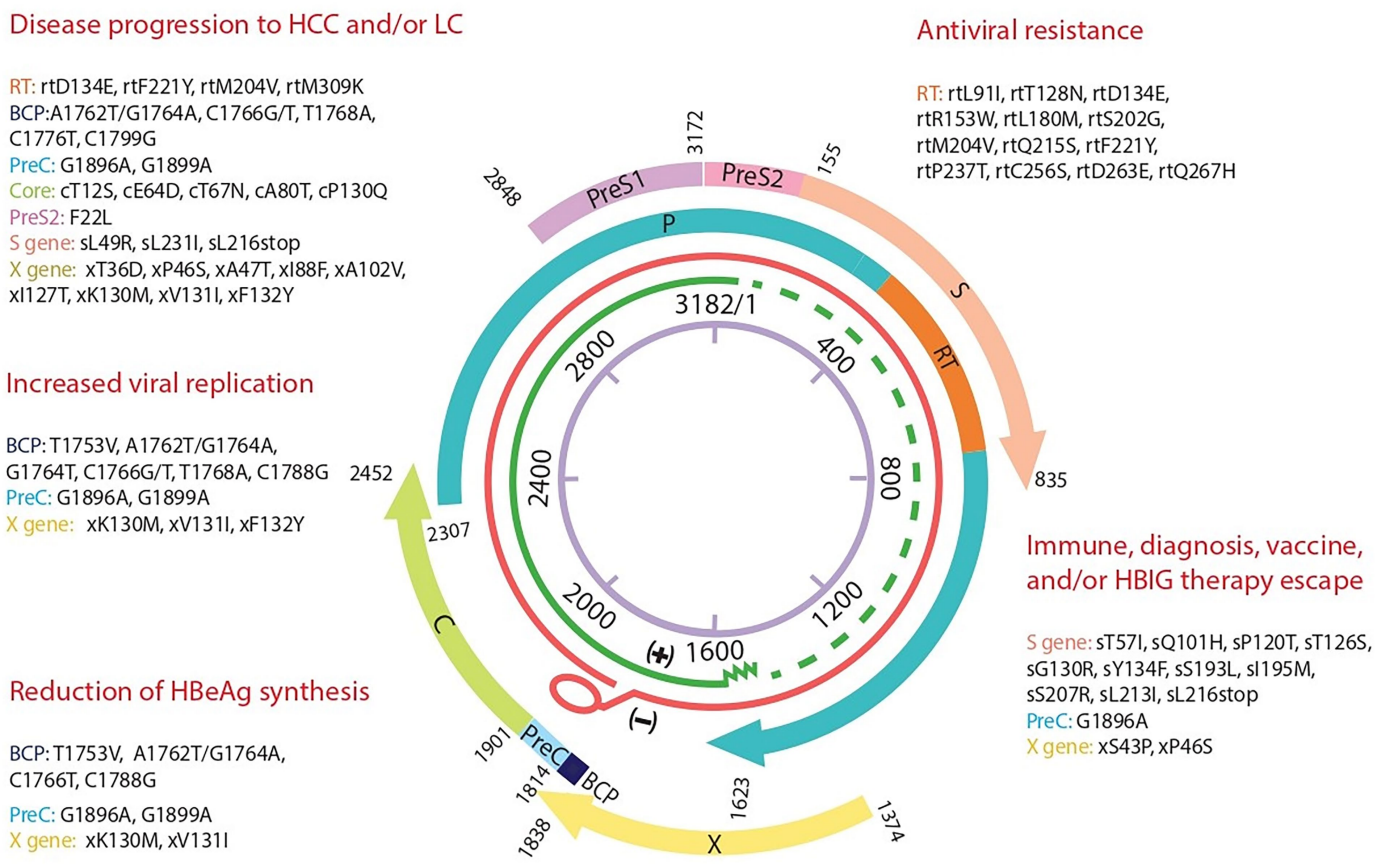
Hepatitis B genome map with summary of the most significant mutations and their clinical impact detected in 5 Tunisian HBV patients. A representation of the four overlapping genes encoding the polymerase P, the surface gene S, the capsid gene C, and X gene. The detected significant mutations are grouped depending on their clinical impact based on the literature. BCP, Basal core Promotor; C, core; PreC, precore; S, surface; RT, reverse transcriptase; HBIG, Immunoglobulin.

Mutations that did not fit categories 1 to 4 were classified into “novel amino acid substitutions” and were observed in 12 aa positions. Six variations namely rtE11D, rtH54Y, rtW257Y, rtD263E, rtQ267Y, and rtE271D were found in treatment-naïve patients and six variations namely rtL145M, rtL260F, rtQ267R, rtK270R, rtM309K, and rtN337T occurred during treatment. The aa changes detected in the RT region of the P gene are mentioned in Table 2; Figure 3.

**Table 2:** Amino acid substitutions detected within the RT region sequences of the five HBV Chronic infected patients with their reported antiviral resistance.

Amino acid substitution	Mutation category	Patients		Drug resistance	Change in overlapping genes	References
		Treatment naïve	During treatment			
E11D	Novel mutation	P4	–	Unknown	N.C	Horikita et al. (1994)
H54Y	Novel mutation	P4/P5	P3	Unknown	N.C	Yang et al. (2002)
N76D	Novel mutation	–	P2	Clinical failure of famciclovir	N.C	Günther et al. (1999); Delaney et al. (2001); Schildgen (2007)
L91I	Secondary/compensatory	P2	P2	LMV ETV	N.C	Ciftci et al. (2014); Mahabadi et al. (2013); Karatayli et al. (2012); Yamani et al. (2017)
R110G	Pre-treatment	P1	P1	Potential resistance	N.C	Ciftci et al. (2014); Biswas et al. (2013); Azarkar et al. (2018)
T128N	Secondary/compensatory	P2	P2	LMV	sP120T	Torresi et al. (2002a); Locarnini et al. (2003)
D134E	Putative	–	P3	TDF	sT126S	Liu et al. (2010); Park et al. (2019); Zheng et al. (2012); Choi et al. (2018)
L145M	Novel mutation	–	P4	Unknown	N.C	Katsoulidou et al. (2009)
Q149K	Secondary/compensatory	P1/P4/P5	P1/P4	Unknown	N.C	Germer et al. (2003)
R153W	Putative	P1/P4/P5	P1/P4	TDF	N.C	Mokaya et al. (2020); Ismail et al. (2011); Li et al. (2012); Mokaya et al. (2019); Olusola et al. (2021)
L180M	Secondary/compensatory	–	P2/P3	LMV, ETV, LdT, TDF	N.C	He et al. (2015); Choi et al. (2018); Yang et al. (2005)
S202G	Primary	–	P2/P3	LMV, ETV	sS193L	Villet et al. (2007); Mukaide et al. (2010)
M204V	Primary	–	P2/P3	LMV, LdT, ETV, TDF	sI195M	He et al. (2015); Li et al. (2005)
Q215S	Secondary/compensatory	–	P2	LMV, ADV	sS207R	Shaw et al. (2006); Moriconi et al. (2007); Amini-Bavil-Olyaee et al. (2009); Liu et al. (2009); Wang et al. (2017)
F221Y	Secondary/compensatory	–	P2	ADV	sL213I	Pollicino et al. (2009); Li et al. (2017); Choi et al. (2018)
P237T	Secondary/compensatory	P1/P4/P5	P1/P4	ADV	N.A	Pollicino et al. (2009)
C256S	Putative	–	P1	LMV, TDF	N.A	Ciftci et al. (2014); Mokaya et al., (2020); Ciancio et al. (2004)
W257Y	Novel mutation	P2/P3	P2/P3	Unknown	N.A	Ismail et al. (2011)
L260F	Novel mutation	–	P4	Unknown	N.A	Not reported
D263E	Pre-treatment	P5	–	Potential partial resistance to TDF	N.A	Bakhshizadeh et al. (2015)
I266R	Pre-treatment	P1	P1	Unknown	N.A	Westland (2003)
Q267H/R/Y	H:Secondary/compensatory	P4/P5	P4	H: LMV, LdT	N.A	Qin et al. (2013b)
	R:Novel mutation	–	P2	Unknown	N.A N.A	Qin et al. (2013a)
	Y:Novel mutation	P1	P1	Unknown		Not reported
K270R	Not reported	-	P2	Unknown	N.A	Quiros-Roldan et al. (2008)
E271D	Novel mutation	P4	P4	Unknown	N.A	Quiros-Roldan et al. (2008)
M309K	Novel mutation	–	P3	Unknown	N.A	Wu Y et al. (2014)
N337T	Not reported	–	P2	unknown	N.A	Boyd et al. (2019)

P1–P5 = patients 1–5. NC, no change; mutation is silent in the surface antigen reading frame. NA, not applicable; polymerase substitution is downstream of the surface antigen reading frame. ADV, Adefovir dipivoxil; ETV, Entecavir; LdT, Telbivudine; LMV, Lamivudine; TDF, Tenofovir disoproxil fumarate.

#### Mutational analysis of the pre-S/S coding regions

A total of 35 aa substitutions were observed in the whole S gene ranging between 3 and 18 mutations per patient, most of them (*n* = 16) were located in the S region. In the pre-S1 and pre-S2 regions, *n* = 9 and *n* = 10 substitutions were observed, respectively. Mutations detected in the S gene are summarized in Table 3; Figure 3. Out of the 16 aa changes in the S region, 10 were clustered in HBsAg epitopes including B-cells epitopes (aa100-160) as follows: 6 (sN3T, sL42R, sL49R, sT57I, sC76Y, and sQ101H) in HBs1 (upstream of aa120); 1 (sP120T) in HBs2 (aa120–123) and 3 (sT126S, sG130R and sY134F) in HBs3 (aa124–137). Furthermore, the major hydrophilic region (MHR, aa99–169) of the S region had accumulated 5 aa variations of which three were within the HBsAg “a” determinant region (aa124–147).

**Table 3:** Amino acid substitutions within the HBV surface gene sequences from the studied patients with their impact.

Region	Cell subsets	Amino acid substitution	Patients		Effects	References
			Treatment Naive	During treatment		
Pre S1 region		A28T	P1/P5	P1	Unknown	Taghiabadi et al. (2019)	
		A28N	P3	P3	Unknown	Feeney et al. (2013)	
		T40P	P5	–	Unknown	Pourkarim et al. (2014)	
		H60D P78T S85C	P3	–	Unknown	Not reported	
		I74L	P2/P3	P2/P3	Unknown	Mondal et al. (2015)	
		S90L	P1	P1	Unknown	Not reported	
		N103D	P5	P2	Unknown	Mondal et al. (2015)	
Pre S2 region		T11N	P3	P3	Unknown	Pourkarim et al. (2014)	
		R16K	P2	–	Unknown	Pourkarim et al. (2014)	
		R18K	P1	P1/P2	Unknown	Lago et al. (2014)	
		F22L	P1	P1/P2	Association with HCC progression	Gopalakrishnan (2013); Chaudhuri et al. (2004)	
		N33D	–	P2	Unknown	Kim et al. (2013)	
		A39V	P2/P3/P4	P2/P3	Unknown	Pollicino et al. (2007)	
		P41H	P2/P3/P5	P2/P3	Unknown	Pollicino et al. (2007)	
		I42T	P3	P2/P3	Unknown	Kim et al. (2010)	
		F46S	P2	–	Unknown	Olinger et al. (2007)	
		P52L	P3	–	Unknown	Pollicino et al. (2007)	
S region	Other	N3T	P4	–	Unknown	Not reported	
	T-helper (CD4) epitope (aa21-65)	L42R	-	P2	Unknown	Chaouch et al. (2016)	
		L49R	P3	–	Association with LC progression	Chaouch et al. (2016)	
		T57I	P5	–	Reduced HBsAg antigenicity	Duda (2020)	
	Other B-cell epitope (aa 100–160)	C76Y	P5	–	Unknown	Wei et al. (2011)	
		Q101H	P2	P2	Immune escape	Tokgöz et al. (2018)	
		P120T	P2	P2	HBIG therapy escape Misdiagnosis Vaccine escape Reduced HbsAg secretion	Amini-Bavil-Olyaee et al. (2010); Bahramali et al. (2008)	
		T126S	–	P3	HBIG therapy escape Vaccine escape Misdiagnosis	Moerman et al. (2004); Sitnik et al. (2004)	
		G130R	P5		Immune escape	Kwei et al. (2013); Tokgöz et al. (2018)	
		Y134F	P2	–	Immune escape	Chaouch et al. (2016); Coppola, (2015)	
	T-helper (CD4) epitope (aa 186–197)	S193L	–	P2	Vaccine escape	Aydın et al. (2019); Suntur et al. (2019)
		I195M	–	P2/P3	Vaccine escape Misdiagnosis Reduced *in vitro* affinity to anti-HBs antibodies.	Colagrossi et al. (2018); Torresi et al. (2002b); Araujo et al. (2008)
	CTL (CD8) epitope (aa 206–215)	S207R	–	P2	Immune escape	Hosseini et al. (2019)
		P211R	–	P4	Unknown	Choga et al. (2020)
		L213I	–	P2	Immune escape	Hosseini et al. (2019); Datta et al. (2014)
	T-helper (CD4) epitope (aa 215–223)	L216stop	P3	–	Truncated HbsAg protein Misdiagnosis Reduced HbsAg secretion Association with HCC progression	Araujo et al. (2008); Hosseini et al. (2019)

P1–P5 = Patients 1–5. HCC, hepatocellular carcinoma; LC, liver cirrhosis.

In addition, nine mutations out of the 16 substitutions in the S gene occurred in different CD4 and CD8 recognition epitopes with the following distribution: 6 aa changes (sL42R, sL49R, sT57I, sS193L, sI195M, and sL216*) in T-helper CD4 epitopes (aa21–65/aa186-197/aa215–223) and 3 (sS207R, sP211R, sL213I) within cytotoxic T lymphocyte CD8 epitopes (aa206-215).

Immune escape mutations (sQ101H, sG130R, sY134F, sS207R, and sL213I) were detected in patients 2 and 5, HBsAg vaccine escape mutations (sP120T, sT126S, sS193L, and sI195M) were found in patients 2 and/or 3, HBIG immunotherapy escape mutations (sP120T and sT126S) were detected in patients 2 and 3, respectively, and misdiagnosis mutations (sP120T, sT126S, sI195M, and sL216stop) were observed in patients 2 and/or 3. Other mutations such as sN3T, sL42R, sC76Y, and sP211R are either not reported or with unknown impacts are also detected in our study.

#### Mutational analysis of the basal core promotor BCP, precore, and core coding regions

The analysis of the 9 HBV genomic sequences bearing the BCP, pre-C, and Core regions is summarized in Table 4. In the BCP region, 14 different aa mutations were identified over 10 sites ranging between 2 and 8 per patient. Nucleotide mutation G1757A was detected in patients 2, 3, 4, and 5; A1762T, G1764A, and T1753V were detected in patients 1, 3, and 5; C1773T in patients 2 and 3, G1764T/C1766G in patient 2 and C1766T/T1768A in patient 3. The double mutation G1764A/A1762T was found in patients 1, 3, and 5.

**Table 4:** Amino acid/nucleotide substitutions detected within the BCP, recure, and core sequences of the five chronic HBV infected patients with their impact.

Region	Cell subsets	Substitution		Patients		Effects	References
		Amino acid	Nucleotide	Treatment naïve	During treatment		
Basal core promotor		N.A	A1752G	P3	P3	Low viral replication capacity	Quarleri (2014); Ng et al. (2005)
		N.A	T1753V (C/A/G)	P1/P5 (C) P3 (A)	P1 (C) P3 (G)	Increase viral replication Reduction in HbeAg synthesis	Caligiuri et al. (2016); Parekh et al. (2003)
		N.A	G1757A	P2/P3/P4/P5	P2/P3/P4	Protection from liver disease progression to LC and/or HCC	Poustchi et al. (2008); Ducancelle et al. (2013); Mohamadkhani et al. (2011)
		N.A	A1762T	P1/P3/P5	P1/P3	Reduction in HbeAg synthesis May increase viral transcription and replication HbeAg seroconversion Association with liver disease progression to HCC or LC	Quarleri (2014); Chen et al. (2005); Leng et al. (2015); Yan et al. (2015); Fang et al. (2008)
		N.A	G1764A	P1/P3/P5	P1		
		N.A	G1764T	P2	P2	Increase viral replication Increase core promoter activity	Sendi et al. (2005); Poustchi et al. (2008)
		N.A	C1766G	P2	P2	increase core promoter activity Increase viral replication Association with liver disease progression to LC	Sendi et al. (2005); Poustchi et al. (2008); Salarneia et al. (2016)
		N.A	C1766T	P3	P3	Increase viral replication Reduction in HbeAg synthesis Association with liver disease progression to HCC and LC	Tong et al. (2013); Kitab et al. (2012); Nishizawa et al. (2016)
		N.A	T1768A	P3	P3	Increase viral replication Association with liver disease progression to HCC and LC	Yin et al. (2011); Jammeh et al. (2008); Huang et al. (2011)
		N.A	C1773T	P2/P3	P2/P3	Association with liver disease progression to HCC and LC	Ghosh et al. (2012); Yin et al. (2011); Gil-García et al. (2019)
		N.A	C1788G	–	P4	Reduction in HbeAg synthesis -Increase viral replication	Tong et al. (2013)
	N.A	C1799G	P2	P2	Inversely associated with HCC and significantly associated with LC	Chen et al. (2005); Yin et al. (2011)
Precore		W28stop	G1896A	–	P2	Inhibition of HbeAg synthesis Immune escape to anti-Hbe Increase viral replication Association with liver progression to LC and HCC	Kargar Kheirabad et al. (2017); Al-Qahtani et al. (2018); Tong et al. (2007)
		G29D	G1899A	P1/P2/P3	P1/P3	Inhibition of the recognition and cleavage of HbeAg precursor May increase viral replication Association with disease progression to LC and HCC	Thompson et al. (2010); Liao et al. (2012); Ouneissa et al. (2012); Al-Qahtani et al. (2018)
Core	Other	T12S	A1934T	P1	P1	Association with disease severity	Datta et al. (2014); Saha et al. (2014)
	CTL (CD8) epitope (aa 18–27)	S21T	T1961A	P1	P1	Unknown	Sominskaya et al. (2011)
	Other	D29H	G1985C	P5	-	Unknown	Not reported
	T-helper (CD4) epitope (aa 35–45)	E40D	A2020T	P1/P4/P5	P1/P4	Unknown	Pollicino et al. (2007)
		E40Q	G2018C A2020T	–	P3	Unknown	Homs et al. (2012)
	CTL (CD8) epitope (aa 50–69) + T-helper (CD4) epitope (aa 48–69)	E64D	A2092C	P3/P4	P2/P4	Association with disease progression to LC and HCC Reduction in T-cell proliferation in association with T67N	Pollicino et al. (2007); Al-Qahtani et al. (2018); Homs et al. (2011)
		T67N	C2100A	P4	P4	Same effects as E64D	Datta et al. (2014); Saha et al. (2014); Pollicino et al. (2007); Homs et al. (2011)
		A69G	C2106G	P4	P4	Unknown	Sominskaya et al. (2011)
	CTL (CD8) epitope (aa 74–83)	V74G	T2121G	P2/P3	P3	Reduction in HBe and HBc antigenicity	Pollicino et al. (2007); Homs et al. (2012)
		V74S	G2120A T2121G	–	P2	Unknown	Not reported
	B-cell epitope (aa 76–89)	P79Q	C2136A	P1	P1	Reduction in HBe and HBc antigenicity	Pollicino et al. (2007); Huang et al. (2014)
		A80T	G2138A	P2/P3	P2/P3	Truncated HBcAg protein → Negativity for anti-HBc. Reduction in HBe and HBc antigenicity. Association with disease progression to HCC or LC	Pollicino et al. (2007); Bajpai et al. (2017); Al-Qahtani et al. (2018)
		A80S	G2138T	P1	P1	Unknown	Not reported
		V85I	G2153A	–	P2	Unknown	Pollicino et al. (2007)
	Other	M93V	A2177G	P3	-	Unknown	Al-Qahtani et al. (2018)
	B-cell epitope (aa 105–116)	I116V	A2246G	P1	P1	Unknown	Pollicino et al. (2007)
	B-cell epitope (aa 130–135)	P130Q	C2289A	–	P2	Association with disease progression to HCC or LC	Datta et al. (2014); Pollicino et al. (2007)
	CTL (CD8) epitope (aa 141–151)	R151Q	G2352A	P1	P1	Unknown	Not reported
	Other	G153C	G2357T	–	P2	Unknown	Wu J et al. (2014)
		S155T	T2363A	–	P2	Unknown	Pollicino et al. (2007)
		P156S	C2366T	P1	P1	Unknown	Not reported
		R166P	C2366T	–	P4	Unknown	Not reported

P1–P5 = Patients 1–5. HCC, Hepatocellular carcinoma; LC, liver cirrhosis.

In the precore region, 2 mutations were identified: G1896A (patient 2) and G1899A (patients 1, 2, and 3). Whereas, 22 mutations were observed in the core region ranging between 2 and 8 per patient. Among them, several amino acid substitutions were found within different antigen immunogenic epitopes. In particular, 4 aa changes were detected in the T-helper CD4 epitopes (aa 35–45 and 48–69; cE40D/Q, cE64D, cT67N, cA69G), 5 in the B-cell epitopes (aa76–89, 105–116, 130–135; cP79Q, cA80T/S, cV85I, cI116V and cP130Q) and 7 in the CTL CD8 epitopes (aa 18–27, 50–69, 74–83, 141–151; cS21T, cE64D, cT67N, cA69G, cV74S/G, cA80T/S, and cR151Q). The detected nucleotide/aa mutations found in the C gene are presented in Table 4; Figure 3.

#### Mutational analysis in the X coding region

In total, 30 aa substitutions were found in the X gene region ranging between 4 and 14 aa substitutions per patient of which 29 were before treatment beginning and only 1 was during it. Among them 10 variations were detected in the B-cell epitope (aa 29–48) namely: xL34I, xT36D/G, xS38P, xS39P, xP40S, xS41P, xL42P, xS43P, xP46S and xA47T. Four mutations; xK95N, xL98I, xA102, and xT105M; were detected within the T-helper CD4 epitope (aa 91–105) and 2 substitutions (xD119N and xL123W) were detected in CTL CD8 epitope (aa115–123).

As BCP overlaps partially with the HBx coding sequence, mutations at nucleotide positions T1753C/A/G, T1762T, G1764A/T, and C1788G; induce amino acid changes xI127T/D/G, xK130M, xV131I/L and xH139D near the C-terminus of the HBx protein, respectively.

The amino acid substitutions detected in the HBV X gene and their impact are summarized in Table 5; Figure 3.

**Table 5:** Amino acid/nucleotide substitutions detected within the X gene sequences of the patients with their reported effects.

Cell subsets	Aa substitution	Nucleotide mutation	Patients		Effects	References
			Treatment naïve	During treatment		
Other	C26S	T1449A	P2	P2	Unknown	Not reported
	C26R	T1449C	P3	P3	Unknown	Pollicino et al. (2007)
B-cell epitope (aa 29–48)	L34I	C1473A	P5	–	Unknown	Not reported
	T36D	A1479G C1480A	P1/P4	P1/P4	Association with HCC progression	Pollicino et al. (2007); Sominskaya et al. (2011); Javanmard et al. (2020)
	T36G	A1479G C1480G	P5	–	Unknown	Not reported
	S38P	T1485C	P1	P1	Unknown	Mani et al. (2019)
	S39P	T1488C	P5	–	Unknown	Pollicino et al. (2007)
	P40S	C1491T	P1/P4	P1/P4	Unknown	León et al. (2005)
	S41P	T1494C	P5	–	Unknown	Xu et al. (2007)
	L42P	T1498C	P2/P3	P2/P3	Unknown	Not reported
	S43P	T1500C	P1/P4/P5	P1/P4	Immune escape (B-cell epitope affected)	Putri et al. (2019); Wang et al. (2012); Li et al. (2018)
	P46S	C1509T	P2/P3	P2/P3	Immune escape (B-cell epitope affected) Association with HCC progression	Pollicino et al. (2007); Li et al. (2018)
	A47T	G1512A	P2/P3/P5	P2/P3	Association with HCC progression	Al-Qahtani et al. (2017); Artarini et al. (2016)
Other	T82S	A1617T	P2	P2	Unknown	Not reported
	H86R	A1630G	P5	–	Unknown	Huang et al. (2014)
	I88F	A1635T	P2	P3	Association with HCC progression	Javanmard et al. (2020); Pollicino et al. (2007)
	I88C	A1635T T1636G	P3	P2	Unknown	Abdel Hamid and Salama (2018)
T-helper (CD4) epitope (aa 91–105)	K95N	G1658C	P1	P1	Unknown	Not reported
	L98I	C1665A	P1	P1	Unknown	Pollicino et al. (2007)
	A102V	C1678T	P2/P3	P2/P3	Association with HCC progression	Ghosh et al. (2012); Mani et al. (2019); Pollicino et al. (2007)
	T105M	C1687T	P3	P3	Unknown	Mani et al. (2019)
CTL (CD8) epitope (aa 115–123)	D119N	G1728A	P3	–	Unknown	Zhu et al. (2008)
	L123W	T1741G	P3	–	Unknown	Not reported
Other	I127T	T1753C	P1/P5	P1	Association with HCC progression Promote transactivation and increase anti-proliferative activity	Al-Qahtani et al. (2017); Artarini et al. (2016); Lin et al. (2005); Elkady et al. (2008)
	I127D/G	T1753A/G	P3 (D)	P3 (G)	Unknown	Not reported
	K130M	A1762T	P1/P3/P5	P1/P3	Increase viral replication and cell invasion Decrease the expression of HBeAg Association with HCC progression	Mani et al. (2019); Lin et al. (2005); Yuan et al. (2009)
	V131I	G1764A	P1/P3/P5	P1	Increase viral replication and cell invasion Decrease the expression of HBeAg Association with disease progression	Al-Qahtani et al. (2017); Mani et al. (2019); Lin et al. (2005); Kim et al. (2016)
	V131L	G1764T	P2	P2	Unknown	Pollicino et al. (2007)
	F132Y	T1768A	P3	P3	Increase viral replication and cell invasion Association with HCC progression	Pollicino et al. (2007); Al-Qahtani et al. (2017); Mani et al. (2019)
	H139D	C1788G	–	P4	Unknown	Not reported

P1–P5 = Patients 1–5. HCC, Hepatocellular carcinoma.

## Discussion

In the present study, we have succeeded to generate the whole HBV genome by amplifying 3 overlapping PCR products covering the entire genome (3.2 kb) using Sanger technology. This technology remains of great importance despite the transition of most laboratories to Next generation sequencing (NGS) technologies. In fact, for small genomes, such as HBV, the Sanger technology is cost effective and more efficient for low viral loads < 10^3^ IU/ml.

The whole genome was assembled for the 5 patients before the treatment and for 4 patients during the treatment. HBV genome was used for genotyping as well as to study the mutational profile in all the genes (S, P, X, and C) in order to give scientific proof of antiviral treatment resistance.

Genotyping showed that genotype D was detected in the 5 studied patients. This genotype was previously described as a predominant HBV genotype in Tunisia and the Maghreb region as well as in the Middle East with a low co-circulation rate of genotype E (Ayed et al., 2007; Ezzikouri et al., 2008; Ouneissa et al., 2013).

Subgenotypes D1 and D7, found in the present study, were previously described as the most prevalent subgenotypes circulating in Tunisia (Meldal et al., 2009). However, subgenotype D8 is to our knowledge detected for the first time in Tunisia. This subgenotype has been firstly detected in Niger and has been described as a recombinant strain between genotypes D and E (Chekaraou et al., 2010). The recombination analysis of the detected D8 strain, using the NCBI viral genotyping tool, was in line with the previous findings. Further studies are needed on larger population size to estimate the prevalence of this subgenotype in Tunisia.

In the second part of the present study, we have analyzed the mutational profile of all HBV genes P, S, C, and X.

The mutational profile of the RT region in the P gene showed high genetic variability with 28 different mutations. Before the treatment, 14 aa mutations were detected of which patient 2 had already 2 secondary/compensatory substitutions: rtL91I and T128N, described to be a resistance mutation to ETV and/or to LMV, respectively (Torresi et al., 2002a; Mahabadi et al., 2013; Ziaee et al., 2016). For the remaining patients, four mutations were detected and reported to be resistant to at least one of the following antivirals: rtQ267H in patients 4 and 5 to LMV and LdT; rtP237T in patients 1, 4, and 5 to ADV; rtR153W in patients 1, 4, and 5 in addition to rtD263E in patient 5 potentially to TDF (Pollicino et al., 2009; Qin et al., 2013b; Bakhshizadeh et al., 2015; Mokaya et al., 2020). The eight remaining substitutions were not previously described to have an impact on antiviral treatment.

During the treatment, 14 additional aa substitutions occurred. The most significant ones were rtM204V, rtL180M, and rtS202G detected in patients 2 and 3. Indeed, it has been described that the rtM204V substitution is usually associated with the compensatory mutation rtL180M, which restores the replication capacity of rtM204V mutants (Tenney et al., 2004). Thus, the pattern rtL180M, rtS202G, and rtM204V act synergistically not only to increase viral load but also to reduce treatment susceptibility and confer cross-resistance to ETV, TDF, LMV, and LdT (Kamiya, 2003; Li et al., 2005; He et al., 2015; Mokaya et al., 2020). Other emerged aa variations have been detected in our patients and previously described as resistance mutations that reduce the affinity and susceptibility to antiviral drugs namely rtQ215S and rtC256S to LMV; rtQ215S and rtF221Y to ADV; rtD134E, rtQ215S, and rtC256S to TDF (Moriconi et al., 2007; Amini-Bavil-Olyaee et al., 2009; Liu et al., 2009; Pollicino et al., 2009; Ciftci et al., 2014; Park et al., 2019; Mokaya et al., 2020).

Thus, our results support the need to introduce HBV genome sequencing as a pre-treatment diagnosis to predict potential resistance to available antiviral molecules, as well as to monitor the evolution of treatment response.

In addition, we have studied the mutational profile in the preS1, preS2, and S genes. As the coding sequence of the HBsAg is completely overlapped with the RT domain of the HBV polymerase, some mutations occurring in the RT region may lead to the emergence of escape mutants in the S region and vice versa. Thus, rtT128N, rtD134E, rtS202G, rtM204V, rtQ215S and rtF221Y substitutions observed in the RT region result in sP120T, sT126S, sS193L sI195M, sS207R and sL213I in the HBsAg gene, respectively. These mutations in addition to sT57I, sQ101H, sG130R, sY134F, and sL216stop could alter the antigenicity of HBsAg and reduce its expression and/or recognition by antibodies. Therefore, they could induce immune, vaccine, HBIG therapy, and/or diagnosis escape as well as influence HBsAg expression and treatment efficacy (Moerman et al., 2004; Sitnik et al., 2004; Bahramali et al., 2008; Amini-Bavil-Olyaee et al., 2010; Coppola, 2015; Ziaee et al., 2016; Rendon et al., 2017; Tokgöz et al., 2018; Aydın et al., 2019; Hosseini et al., 2019; Duda, 2020).

Regarding the mutational profile of BCP (nt 1,742–1,849), precore (nt 1,814–1,900), and core regions that code HBeAg and HBcAg proteins, the double mutants A1762T/G1764A, G1764T/C1766G and C1766T/T1768A, as well as the single mutations A1752G, T1753V (C/A/G), C1766T and C1788G, detected in BCP region, have been reported to enhance viral replication and/or reduce HBeAg synthesis by suppressing the transcription of the pre-C region (Parekh et al., 2003; Sendi et al., 2005; Poustchi et al., 2008; Tong et al., 2013; Caligiuri et al., 2016; Lazarevic et al., 2019). The single nucleotide mutations G1896A and G1899A in the precore region have been suggested to be mutational hotspots occurring most frequently in genotype D and were previously reported in Tunisian studies with an occurrence alone or in association (Triki et al., 2000; Bahri et al., 2006; Ayed et al., 2007; Poustchi et al., 2008; Ouneissa et al., 2012). These mutants result in a stop codon at position W28* and a substitution at position G29D, respectively, leading to the production of a truncated precore protein and then the abolition of HBeAg expression (Kobayashi et al., 2003; Thompson et al., 2010; Ducancelle et al., 2016). These variations are the major immune escape mutants of HBV as HBeAg is the main target for both cellular and humoral immune responses leading to a higher risk of liver HCC and LC progression (Tong et al., 2005; Liao et al., 2012; Suppiah et al., 2015; Pahal et al., 2016). In addition, precore mutants impose serious consequences on the treatment and enhance viral replication (Ouneissa et al., 2012; Kargar Kheirabad et al., 2017; Boyd et al., 2018).

Concerning the core mutations, cT67N within the T-helper CD4 epitope might be able to escape the host immune response (Datta et al., 2014; Saha et al., 2014). Moreover, cV74G, cP79Q, and cA80T mutations are known to reduce both HBe and HBc antigenicity (Pollicino et al., 2007; Huang et al., 2014). In addition, cA80T has resulted in the production of altered and truncated HBcAg protein leading potentially to abnormal immune reaction and negativity of anti-HBc (Bajpai et al., 2017).

In the last part of this study, we studied the mutational profile in the X gene. Substitutions xS43P and xP46S located in the B-cell epitope were detected in our study and have been suggested to be related with immune escape (Putri et al., 2019). Mutations xP46S, xA47T, xI88F, xA102V, xI127T, xK130M, xV131I, and xF132Y, were previously reported as significant HCC-related HBx mutants alone or combined such as (I127T + K130M + V131I) in patients 1, 3 and 5 and (xK130M + xV131I + xF132Y) in patient 3 (Pollicino et al., 2007; Ghosh et al., 2012; Ali et al., 2014; Al-Qahtani et al., 2017). Moreover, the double mutant xK130M + xV131I has been suggested to exacerbate the host’s immune response, increase viral replication, and lead to a truncated HBx protein (Wungu et al., 2019). In addition, it is associated with the activation of proto-oncogenes and inactivation of the tumor suppressor gene leading to a rapid progression of liver cirrhosis and/or HCC cell invasion and metastasis (Wang et al., 2016).

Several mutations previously reported to be significantly associated with an increased risk of severe liver disease progression to HCC and/or LC progression were also detected in other genes namely (rtD134E/rtF221Y/rtM204V/rtM309k) in the RT region; (sF22L) in the preS2 region; (sL49R, sL213I and sL216*) in the S region; (C1766T/T1768A double mutant, C1773T, C1799G, and C1766G) in the BCP region; and (cT12S/cE64D/cT67N/cA80T/cP130Q) in the core region of the C gene. These HCC-related mutations could be used as markers of HCC evolution in particular rtF221Y mutant which has been indicated as an independent risk factor for poor overall survival (Jammeh et al., 2008; Yin et al., 2011; Kitab et al., 2012; Zheng et al., 2012; Gopalakrishnan, 2013; Tong et al., 2013; Datta et al., 2014; Chaouch et al., 2016; Nishizawa et al., 2016; Kim et al., 2017; Li et al., 2017; Al-Qahtani et al., 2018; Choi et al., 2018; Hosseini et al., 2019). In contrast, the early development of G1757A in the BCP reduces the oncogenic potential of HBV suggesting that it might be a protective biomarker in chronic hepatitis B (Poustchi et al., 2008; Mohamadkhani et al., 2011; Ducancelle et al., 2013).

In addition to the commonly mentioned substitutions in all genes (P, S, C, and X), several nucleotide/amino acid substitutions have been detected in our patients (see Tables 2–5) but have never been reported previously or have been reported with unknown impact. Therefore, further studies are necessary to better understand and elucidate the effect of these mutations on HBV treatment, antigenicity, and disease evolution.

## Conclusion

In conclusion, we would propose the whole genome sequencing as a pre-treatment diagnosis to predict potential resistance to available antiviral molecules, as well as to monitor the evolution of treatment response and prevent progression to cirrhosis or hepatocellular carcinoma. Thus, this could contribute to guiding national efforts to optimize relevant HBV treatment management in order to achieve the global hepatitis elimination goal by 2030.

## Data availability statement

The datasets presented in this study can be found in online repositories. The names of the repository/repositories and accession number(s) can be found in the article/supplementary material.

## Ethics statement

Ethical review and approval was not required for the study on human participants in accordance with the local legislation and institutional requirements. Written informed consent for participation was not required for this study in accordance with the national legislation and the institutional requirements.

## Author contributions

ZB, AC, HTr, MG, SA, LH, and MM: conceptualization. ZB, HTo, AS, WH, WK, and LY: methodology. ZB, AC, and HTr: validation. ZB and AC: formal analysis. ZB, AC, HTo, AS, WH, WK, and LY: investigation. ZB, AC, MG, SA, LH, and MM: data curation. ZB and KA: writing—original draft preparation. AC and HTr: editing and reviewing. All authors contributed to the article and approved the submitted version.

## Funding

This work was funded by the Tunisian Ministry of Higher Education [Programme d’Encouragement des Jeunes Chercheurs PEJC, 1ère Edition (2018; project code: 18PJEC07-09)], the Research Laboratory LR20IPT02: “Virus, Vectors and Hosts: One Health approach and technological innovation for a better health,” and the Clinical Investigation Center (CIC).

## Conflict of interest

The authors declare that the research was conducted in the absence of any commercial or financial relationships that could be construed as a potential conflict of interest.

## Publisher’s note

All claims expressed in this article are solely those of the authors and do not necessarily represent those of their affiliated organizations, or those of the publisher, the editors and the reviewers. Any product that may be evaluated in this article, or claim that may be made by its manufacturer, is not guaranteed or endorsed by the publisher.

## Footnotes

^1^https://blast.ncbi.nlm.nih.gov/Blast.cgi.

^2^ https://mafft.cbrc.jp/alignment/server/

^3^ http://iqtree.cibiv.univie.ac.at

^4^ http://tree.bio.ed.ac.uk/software/figtree/

^5^ https://www.ncbi.nlm.nih.gov/projects/genotyping/formpage.cgi

## Abbreviations

ADV: Adefovir
aa: Amino acid
DNA: Deoxyribonucleic acid
ETV: Entecavir
HBcAg: hepatitis B core antigen
HBeAg: hepatitis B e antigen
HBIG: Hepatitis B immunoglobulin
HBsAg: Hepatitis B surface antigen
HBV: Hepatitis B virus
HCC: Hepatocellular carcinoma
LdT: Telbivudine
LMV: Lamivudine
MHR: Major hydrophilic region
NCBI: National Center for Biotechnology Information
NGS: Next Generation Sequencing
ORF: open reading frame
PCR: Polymerase chain reaction
RT: reverse transcriptase
TDF: tenofovir disoproxil fumarate
